# Split tolerance permits safe Ad5-GUCY2C-PADRE vaccine-induced T-cell responses in colon cancer patients

**DOI:** 10.1186/s40425-019-0576-2

**Published:** 2019-04-23

**Authors:** Adam E. Snook, Trevor R. Baybutt, Bo Xiang, Tara S. Abraham, John C. Flickinger, Terry Hyslop, Tingting Zhan, Walter K. Kraft, Takami Sato, Scott A. Waldman

**Affiliations:** 1 Department of Pharmacology and Experimental Therapeutics, Thomas Jefferson University, 1020 Locust Street, JAH 368, Philadelphia, PA 19107 USA; 2 Department of Biostatistics and Bioinformatics, Duke Cancer Institute, Duke University, Durham, NC 27710 USA; 3 Department of Medical Oncology, Thomas Jefferson University, Philadelphia, PA 19107 USA

**Keywords:** Colorectal cancer, GUCY2C, Guanylyl cyclase C, Vaccine

## Abstract

**Background:**

The colorectal cancer antigen GUCY2C exhibits unique split tolerance, evoking antigen-specific CD8^+^, but not CD4^+^, T-cell responses that deliver anti-tumor immunity without autoimmunity in mice. Here, the cancer vaccine Ad5-GUCY2C-PADRE was evaluated in a first-in-man phase I clinical study of patients with early-stage colorectal cancer to assess its safety and immunological efficacy.

**Methods:**

Ten patients with surgically-resected stage I or stage II (pN0) colon cancer received a single intramuscular injection of 10^11^ viral particles (vp) of Ad5-GUCY2C-PADRE. Safety assessment and immunomonitoring were carried out for 6 months following immunization. This trial employed continual monitoring of both efficacy and toxicity of subjects as joint primary outcomes.

**Results:**

All patients receiving Ad5-GUCY2C-PADRE completed the study and none developed adverse events greater than grade 1. Antibody responses to GUCY2C were detected in 10% of patients, while 40% exhibited GUCY2C-specific T-cell responses. GUCY2C-specific responses were exclusively CD8^+^ cytotoxic T cells, mimicking pre-clinical studies in mice in which GUCY2C-specific CD4^+^ T cells are eliminated by self-tolerance, while CD8^+^ T cells escape tolerance and mediate antitumor immunity. Moreover, pre-existing neutralizing antibodies (NAbs) to the Ad5 vector were associated with poor vaccine-induced responses, suggesting that Ad5 NAbs oppose GUCY2C immune responses to the vaccine in patients and supported by mouse studies.

**Conclusions:**

Split tolerance to GUCY2C in cancer patients can be exploited to safely generate antigen-specific cytotoxic CD8^+^, but not autoimmune CD4^+^, T cells by Ad5-GUCY2C-PADRE in the absence of pre-existing NAbs to the viral vector.

**Trial registration:**

This trial (NCT01972737) was registered at ClinicalTrials.gov on October 30th, 2013. https://clinicaltrials.gov/ct2/show/NCT01972737

**Electronic supplementary material:**

The online version of this article (10.1186/s40425-019-0576-2) contains supplementary material, which is available to authorized users.

## Introduction

While checkpoint inhibitor and CAR-T cell therapies have initiated a paradigm shift in the management of some cancers [1], there remains an unmet need for improved treatment of colorectal cancer (CRC), the 4th leading cause of cancer and 2nd leading cause of cancer mortality worldwide [2]. At the time of initial diagnosis, about two-thirds of CRC patients undergo surgical resection with curative intent, but 30–50% of these patients experience recurrence and die of their disease. Adjuvant chemotherapy only marginally improves survival in stage III disease, and has no benefit in pN0 (stage I and II; lymph node negative) patients. Moreover, checkpoint inhibitors such as nivolumab and pembrolizumab are effective only in microsatellite instable (MSI) CRC [3], reflecting their high density of mutation-associated neoantigens targeted by effector T cells [4, 5]. In contrast, checkpoint inhibitors are ineffective against microsatellite stable (MSS) CRC which accounts for 85% of cases. These considerations underscore the clinical opportunity for novel therapeutics, particularly immunotherapies, to prevent disease recurrence and improve survival in patients with stage I-III colorectal cancer. In that context, immunotherapeutic paradigms in cancer may be most effective in the prevention of recurrent metastases in patients with minimal residual disease [6]. Thus, emerging tumor vaccine paradigms that promote durable antitumor efficacy without autoimmunity, represent a unique opportunity to improve colorectal cancer outcomes.

Guanylyl cyclase C (GUCY2C), a membrane-spanning receptor synthesizing the second messenger cyclic GMP (cGMP), is selectively expressed by intestinal epithelial cells and a subset of neurons [7–10] and near-universally overexpressed in colorectal cancer. Indeed, GUCY2C has been detected in ~ 1000 CRC specimens, but not in extra-gastrointestinal parenchymal tissues or tumors [7, 11, 12]. Moreover, within intestinal epithelial cells, GUCY2C is localized in apical brush border membranes, placing it outside the mucosal barrier [13]. The anatomical and functional compartmentalization of GUCY2C has been confirmed by RT-qPCR [13, 14], radioligand imaging and biodistribution [13], and immunotoxin [15], vaccine [16–20], and CAR-T cell [21, 22] treatment. Together, intestinal compartmentalization and near-universal expression by primary and recurrent colorectal cancer [14, 23, 24], establish GUCY2C as an attractive target for immunotherapeutic prevention of colorectal cancer recurrence.

Adenovirus (Ad5)-delivered GUCY2C-based vaccines induce antigen-specific CD8^+^ T-cell and antibody responses in syngeneic mice [16–20, 25–27]. Mediated by CD8^+^ T-cells rather than antibodies, these immune responses target colorectal cancer metastases in lung and liver in mouse models of prophylaxis and therapy [16, 18–20, 26, 27]. Immunization with GUCY2C-based vaccines produces memory CD8^+^ T-cell responses that provide durable protection against metastases in mice, modeling vaccination in CRC patients with minimum residual disease [16–18]. Importantly, GUCY2C vaccination provides therapeutic efficacy in the absence of autoimmunity [16–20].

Beyond the safety and efficacy of GUCY2C vaccination, preclinical studies in mice demonstrated that self-tolerance, which limits the production of immune responses to self proteins and subsequent autoimmunity, reduced vaccine-induced CD8^+^ T-cell responses to GUCY2C, and eliminated GUCY2C-specific antibody and CD4^+^ T-cell responses [18–20]. However, self-tolerance in mice did not directly impact GUCY2C-specific CD8^+^ T cells and antibody-producing B cells [18]. Rather, self-tolerance eliminated GUCY2C-specific CD4^+^ T cells, which serve an essential “helper” role in the production of CD8^+^ T-cell and B-cell responses [18]. Thus, self-tolerance is uniquely “split” - eliminating GUCY2C-specific CD4^+^ T cells, while preserving functional pools of CD8^+^ T and B cells which can be activated with GUCY2C-independent CD4^+^ T-cell help [18]. Indeed, inclusion of CD4^+^ T-cell epitopes from influenza hemagglutinin (S1) or the synthetic CD4^+^ T-cell epitope PADRE fully activated GUCY2C-specific CD8^+^ T and B cells, improving vaccine antitumor efficacy > 750%, without autoimmunity [17, 18]. Here, we translate observations of split tolerance to GUCY2C from animal models to humans in a phase I clinical trial establishing selective CD4^+^ T-cell tolerance as a key mechanism influencing cancer vaccine responses in humans, and which may be leveraged to elicit antitumor immunity without autoimmune toxicity.

## Materials and methods

### Study design and treatment

This was a phase I study (ClinicalTrials.gov identifier NCT01972737) of stage I or II (pN0) colon cancer within 3 years of surgery and no clinical or laboratory evidence of local or systemic recurrence. The study protocol and all amendments were approved by the Thomas Jefferson University Institutional Review Board (IRB) and Institutional Biosafety Committee (IBC). The study was conducted in accordance with the protocol, Good Clinical Practice guidelines, the ethical principles outlined in the Declaration of Helsinki, and the NIH Guidelines for Research Involving Recombinant or Synthetic Nucleic Acid Molecules. All patients provided written informed consent to participate.

**Fig. 1:**
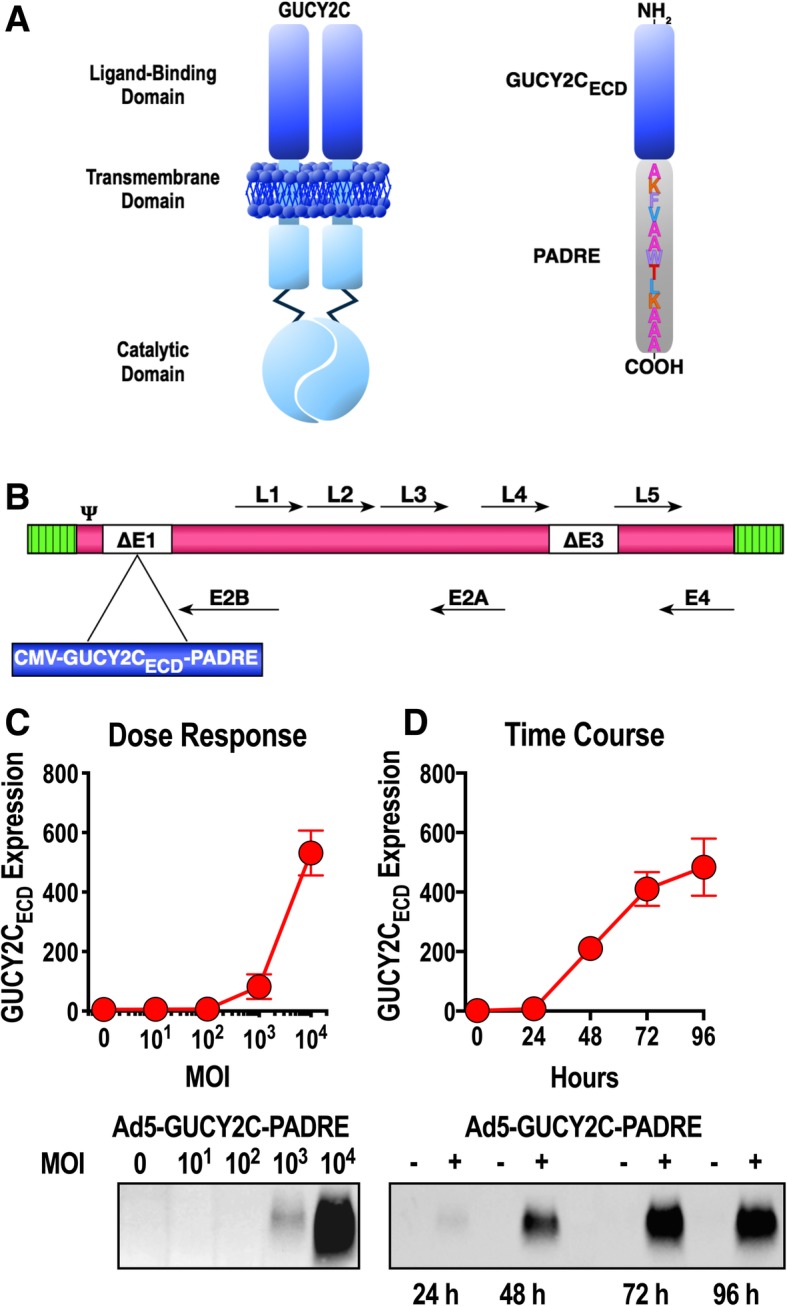
Ad5-GUCY2C-PADRE design and antigen expression. **a** GUCY2C is a membrane-spanning enzyme possessing an extracellular ligand-binding domain and intracellular cGMP-producing catalytic domain. The extracellular domain (ECD) of GUCY2C was employed in the vaccine design and included the PADRE epitope on its COOH-terminus. **b** GUCY2C_ECD_-PADRE was inserted into the E1 region of E1/E3-deleted Ad5. **c** A549 cells were transduced in duplicate with Ad5-GUCY2C-PADRE at an MOI of 10 to 10,000 for 48 h. GUCY2C_ECD_-PADRE expression was quantified in supernatants by immunoblot analysis. Densitometry was employed to quantify expression. **d** A549 cells were transduced in duplicate with Ad5-GUCY2C-PADRE at an MOI of 10,000 for 24–96 h and GUCY2C_ECD_-PADRE expression was quantified in supernatants by immunoblot analysis. Densitometry (arbitrary units) was employed to quantify expression. Blots in **c** and **d** are representative of two experiments and graphs indicate the mean ± SD from 2 experiments

Ad5 vectors have well-established potency to induce antigen-specific immune responses in animal models and humans, as well as a long and impressive safety record. PADRE is a CD4^+^ T-cell epitope that is active in the context of most human HLA molecules [28] and is required to optimally induce GUCY2C-specific immune responses and efficacy in animal models [17, 18]. Codon-optimized cDNA encoding human GUCY2C residues 1–429 with a C-terminal PADRE epitope (Fig. 1a) was cloned into the E1 region of pAd/CMV/V5 (Life Technologies, Carlsbad, CA) containing E1- and E3-deleted human serotype 5 adenovirus (Ad5; Fig. 1b). Ad5-GUCY2C-PADRE vector used for these studies was produced under GMP conditions at the Baylor College of Medicine in the Cell and Gene Therapy Vector Development Laboratory.

In vitro GUCY2C-expression experiments (dose-response and time-course) were carried out in A549 (ATCC, Manassas, VA) cells. Virus was added to the cultures at the indicated doses and culture supernatants were collected at the indicated time points. Relative GUCY2C levels were quantified in supernatants by western blot using MS7 mouse anti-GUCY2C monoclonal antibody [9, 21, 26] and HRP-conjugated goat anti-mouse secondary antibody (Jackson Immuno, West Grove, PA).

Patients received a single intramuscular injection of 10^11^ viral particles (vp) Ad5-GUCY2C-PADRE. Safety assessment was carried out in-clinic for 30 min and via phone call for 1 week. Patients returned for in-clinic safety assessment and immunomonitoring blood collection on days 30, 90, and 180 days after immunization. This trial employed continual monitoring of both efficacy and toxicity of subjects as joint primary outcomes. All patients completed the study.

### Human subject Immunomonitoring

Venous blood was collected into BD Vacutainer® Glass Serum Tubes for serum collection and BD Vacutainer® CPT™ Mononuclear Cell Preparation Tubes with Sodium Citrate for peripheral blood mononuclear cell (PBMC) isolation. For serum collection, blood samples were incubated 30 min at 37 °C, centrifuged, and supernatants were transferred to cryovials and stored at − 20 °C. PBMCs were collected by centrifugation according to manufacturer’s instructions. PBMCs were then washed with 1x CTL-WASH™ buffer (Cellular Technology Limited, Cleveland, OH), counted using a Muse Cell Analyzer (Millipore, Darmstadt, Germany), and cryopreserved in CTL-Cryo™ ABC freezing medium (Cellular Technology Limited) according to the manufacturer’s instructions. Cryovials were frozen at − 80 °C overnight in a CoolCell® LX Alcohol-free Cryopreservation Container (Biocision, Mill Valley, CA) before long-term storage in LN_2_.

#### GUCY2C-specific antibody quantification by ELISA

Hexahistidine-tagged human GUCY2C_ECD_ (amino acids 1–429) protein was produced in suspension HEK293 cells and purified to > 90% purity by immobilized metal affinity chromatography (GenScript, Piscataway, NJ). Nunc-Immuno PolySorp plates (Nunc, Roskilde, Denmark) were coated for 4 h at room temperature with human GUCY2C_ECD_ protein at 10 μg/mL in coating buffer (Immunochemistry Technologies, Bloomington, MN). Plates were washed and free binding sites were blocked with SynBlock (Immunochemistry Technologies) overnight at room temperature. Serum samples were thawed and titrated in coated, washed plates from 1/20 to 1/2560 in 10% nonfat dry milk and incubated 2 h at room temperature. Plates were washed and bound human antibody was detected with HRP-conjugated goat anti-human antibody (Jackson Immuno) for 2 h at room temperature. Following a final wash, Turbo TMB substrate (ThermoFisher Scientific Pierce, Waltham, MA) was added and the plates incubated for color development, followed by determination of optical absorbance (POLARstar Optima plate reader, BMG Labtech, Cary, NC).

#### T-cell response quantification by ELISpot

Human IFNγ SC Enzymatic ELISpot plates (Cellular Technology Limited) were coated according to the manufacturer’s instructions. PBMC samples were thawed using CTL Anti-Aggregate Wash™ (Cellular Technology Limited) according to the manufacturer’s instructions and 5 × 10^5^ viable cells/well were plated in ELISpot plates in serum-free CTL-Test™ Medium (Cellular Technology Limited) without a rest period. Antigens were prepared and added to PBMCs at the indicated final concentrations in CTL-Test™ Medium (Cellular Technology Limited) with 1% DMSO in all conditions: 1% DMSO (ATCC); 1 μg/mL Ad5 Peptide Mix (PM-HAdV5, JPT Peptide Technologies, Berlin, Germany); 2 and 10 μg/mL human GUCY2C Peptide Mix (custom 15 mer/11 aa overlap library of human GUCY2C_1–429_, JPT Peptide Technologies); 1 μg/mL PADRE (BAP-251, EMC Microcollections, Tübingen, Germany). Plates were incubated overnight at 37 °C/5% CO_2_ followed by development according to the manufacturer’s instructions. Spots were quantified using an ImmunoSpot® S6 Universal Analyzer (Cellular Technology Limited). For CD4/CD8 depletion experiments, CD4^+^ or CD8^+^ T cells were negatively selected from thawed PBMC samples by magnetic-activated cell sorting (MACS; Miltenyi Biotec, Bergisch Gladbach, Germany) prior to ELISpot analysis. Small aliquots of PBMCs or CD4- or CD8-depleted PBMCs were stained with anti-CD4-PerCP (clone S3.5, Invitrogen, Carlsbad, CA) and anti-CD8-Alexa Fluor® 700 (clone 3B5, Invitrogen) and analyzed on a BD LSR II flow cytometer. Analyses were performed using FlowJo software (FlowJo, LLC, Ashland, CA).

#### Ad5 NAb titer quantification

Mouse or human serum samples were heat-inactivated for 1 h at 56 °C and then titered in duplicate from 1/20 to 1/10240 in black 96-well tissue culture plates, 50 μL/well final volume. 10^8^ vp of Ad5-CMV-eGFP virus (Vector Development Lab, Baylor College of Medicine) was added to each well of titered serum (50 μL/well of 2x10^9^ vp/mL). 10^5^ A549 cells (ATCC) were then added to each well (100 μL of 10^6^ cells/mL). For quantification of % neutralization by serum samples, controls included virus with cells alone (0% neutralization) and cells alone (100% neutralization). Plates incubated 41 h at 37 °C/5% CO_2_ before quantification of eGFP fluorescence (490 nm excitation, 510 nm emission) using a POLARstar Optima plate reader (BMG Labtech). Sample fluorescence was normalized to controls described above and titers were determined using nonlinear regression as the serum dilution producing 50% neutralization (Prism v7, GraphPad Software, La Jolla, CA).

### Mouse Ad5 NAb studies

Animal studies were approved by the Thomas Jefferson University Institutional Animal Care and Use Committee (IACUC). All mouse studies employed ~ 10 week old female BALB/c mice (Jackson, Bar Harbor, ME). Females were nulliparous and not pregnant. To establish Ad5 NAb titers, vehicle control (naïve) or control Ad5 (Ad5 NAb High) were administered intramuscularly as two 50 μL injections, one in each of the two hind limbs. Ad5 exposure was repeated 21 days later to establish high Ad5 NAb titers. Two weeks after completing the above Ad5 exposures, serum was collected for Ad5 NAb titer determinations and animals were immunized with 10^8^ IFU Ad5-GUCY2C-S1 [18]. Two weeks later, serum and splenocytes were collected and GUCY2C-specific antibody and GUCY2C- and Ad5- specific CD8^+^ T-cell responses were quantified as previously described [16–20].

### Statistical analysis

#### Human antibody responses

A mixed effect model assuming the interaction between serum dilution (1/20, 1/40, etc.) and immune status (pre-vaccination vs post-vaccination) with random effect of replications was applied and the one-sided comparison of immune status at different dilutions was determined. The titer was identified as the greatest dilution producing a significantly higher signal than pre-vaccination serum at the same dilution.

#### Human T-cell responses

For GUCY2C-specific responses, data obtained from 10 μg/mL GUCY2C was employed for analysis unless pre-vaccination signals with 10 μg/mL GUCY2C were > 50 spots/well, indicating a high level of non-specific activation at that concentration. In that case, data obtained from 2 μg/mL GUCY2C was used for analysis. Modified Distribution Free Resampling (mDFR) algorithms [29] were applied to compare antigen-stimulated (test count) responses to DMSO (control count) at each day, as well as the pairwise comparisons of the antigen-specific changes (DMSO-subtracted) between day 0 (pre-vaccination; control count) and each post-vaccination time point (test count). The difference between the log of the test count and the log of the background control count is referred to as mDFR(eq), while the difference between the log of the test count and twice the log of the background control count is referred to as mDFR(2x). A positive antigen-specific response (antigen vs. DMSO) required that antigen vs DMSO at time point *X* is *P* < 0.05 ***and*** antigen-specific spots at time *X* > 5. A positive vaccine-induced response at time point *X* (antigen-specific response at time X vs time 0) required that antigen vs DMSO at *X* is *P* < 0.05 ***and*** antigen-specific response (antigen minus DMSO) at time *X* vs time 0 is *P* < 0.05 ***and*** antigen-specific spots at time *X* > 5. We refer to a result as strongly significant if the mDFR(2x) *P* < 0.05 and moderately significant if it is not strongly significant, but the mDFR(eq) *P* < 0.05. ELISpot responses in patients following CD4/CD8-depletion were compared by Two-way ANOVA with GraphPad Prism v7. For comparisons of Ad5 NAb High and Low patients, for each antigen (GUCY2C, PADRE, and Ad5), the mean difference of antigen and DMSO between High patients and Low patients was compared. A mixed effect model assuming the interaction between time and Ad5 NAb status (High vs. Low) with random effect of patients was applied and Low vs. High differences between each day and day 0 were determined.

#### Animal models

Responses in animal models were compared by T-test or Two-way ANOVA, as appropriate, with GraphPad Prism v7.

## Results

### Ad5-GUCY2C-PADRE vector

Ad5-GUCY2C-PADRE is composed of an E1/E3-deleted recombinant human type 5 adenovirus expressing the human GUCY2C extracellular domain (ECD; GUCY2C_1–429_) fused on its C-terminus to the universal CD4^+^ T-helper cell epitope PADRE (Fig. 1a and b). Previous studies demonstrated that only the extracellular domain of GUCY2C is a viable vaccine target reflecting the high sequence conservation of the intracellular domains of guanylyl cyclase family members and broad tissue distribution of guanylyl cyclases A, B, and G [20]. GUCY2C_ECD_-PADRE and an upstream CMV promoter were cloned into the E1 region of Ad5 (Fig. 1b). Replication-deficient Ad5-GUCY2C-PADRE vector was produced in HEK293 cells and purified by CsCl ultracentrifugation employing GMP procedures at the Center for Cell and Gene Therapy, Baylor College of Medicine. In vitro studies confirmed dose-dependent (Fig. 1c) and time-dependent (Fig. 1d) expression and secretion of GUCY2C_ECD_-PADRE protein by western blot.

### Ad5-GUCY2C-PADRE safety profile

Ten colorectal cancer patients were enrolled and treated with 10^11^ vp Ad5-GUCY2C-PADRE. Additional file 1: Table S1 describes the baseline patient characteristics. The median age was 65 (49–76) years, patients were primarily Caucasian (80%) and patients were distributed equally between male and female. All patients had stage I or II colorectal cancer previously treated with surgery but not chemo/radio/immuno-therapy. Treatment-related acute toxicity was assessed in the clinic every 10 min for 30 min after injection and by telephone on days 3 and 8 following vaccination. Patients also returned to the clinic 30, 90, and 180 days after vaccination for safety assessment. All patients completed the study. Adverse events (Table 1) were graded according to The Common Terminology Criteria for Adverse Events (CTCAE version 4.0). Mild grade 1/2 toxicities included injection site pain and fever which are anticipated following a viral vector immunization. No grade 3/4 toxicities occurred at any time during the 6-month follow-up period after vaccination. Moreover, clinical laboratory assessments performed on days 30, 90, and 180, including CBC with differential, comprehensive chemistry panel, and antinuclear antibody (ANA) titers, revealed no vaccine-related adverse events. Importantly, no adverse events related to toxicity in GUCY2C-expressing tissues were observed. GUCY2C is a self protein expressed on the luminal surface of small and large intestinal epithelia [7, 8], as well as anorexigenic hypothalamic and midbrain dopaminergic neurons [9, 10]. However, consistent with mechanisms controlling immune compartmentalization [30], preclinical studies of GUCY2C vaccination in mice [16–20] confirmed the absence of Ad5-GUCY2C-PADRE-induced autoimmunity in intestine or brain.

**Table 1:** Treatment-related toxicities occurring during the 6-months following Ad5-GUCY2C-PADRE vaccination

Toxicity	Grade 1/2	Grade 3/4	Total (%)
Chills/Rigor	2	0	2 (20%)
Dizziness	1	0	1 (10%)
Diaphoresis	1	0	1 (10%)
Injection site or arm pain/swelling	2	0	2 (20%)
Aches	1	0	1 (10%)
Fever	1	0	1 (10%)

### Ad5-GUCY2C-PADRE-induced immune responses

**Fig. 2:**
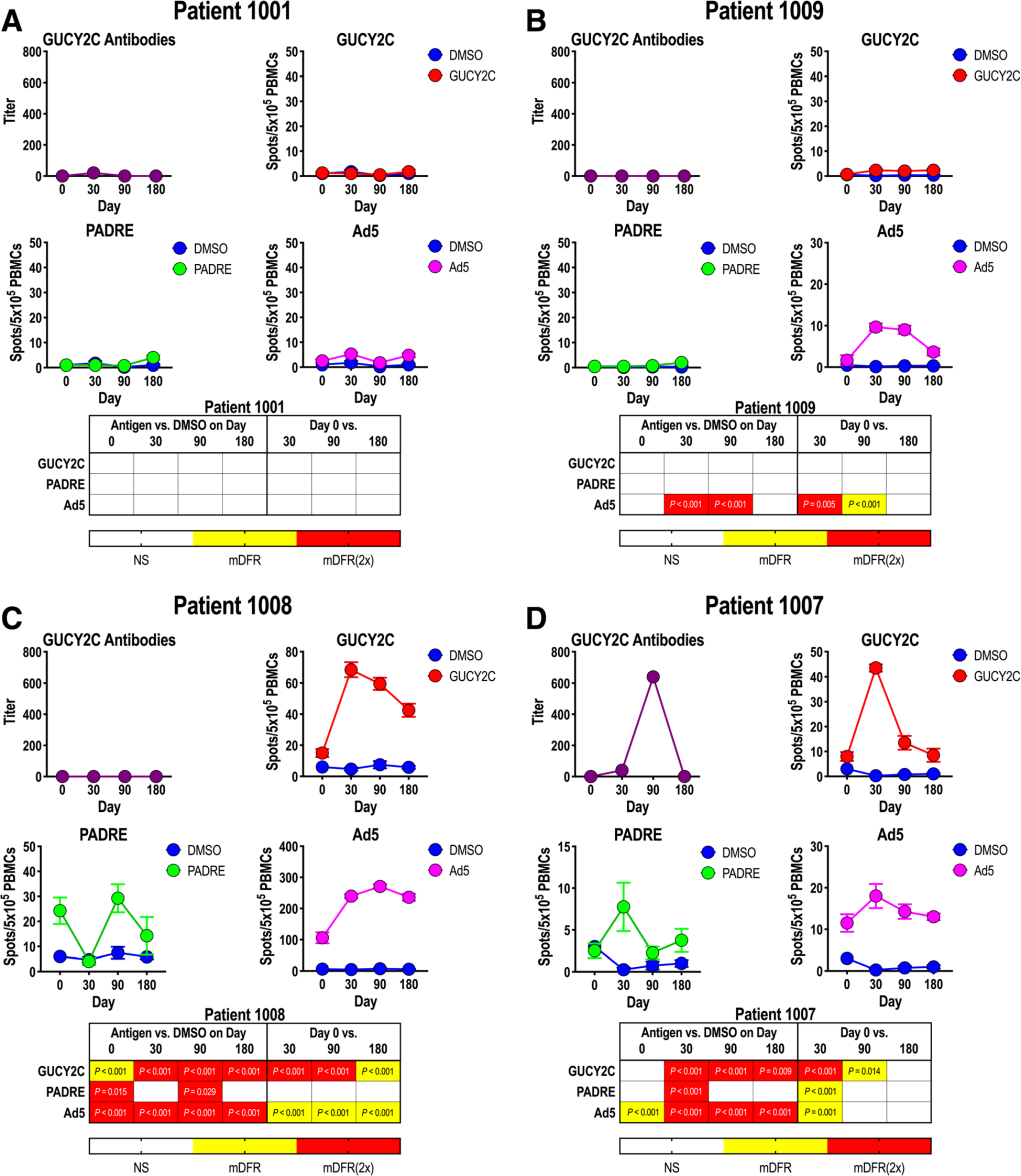
Ad5-GUCY2C-PADRE-induced immune responses. Patient blood samples were collected before (day 0) and 30, 90 and 180 days after Ad5-GUCY2C-PADRE immunization. GUCY2C-specific antibody titers were quantified by ELISA and GUCY2C, PADRE, and Ad5 -specific T-cell responses were quantified by IFNγ-ELISpot. ELISpot assays employed DMSO as an antigen-negative control. The statistical significance for T-cell responses at each time point (compared to DMSO) was determined by modified DFR(eq) or DFR(2x) after Westfall–Young max-T correction, and *p*-values < 5% are shown in yellow [mDFR(eq)] or red [mDFR(2x)], respectively. The statistical significance of T-cell responses obtained for each post-vaccination time point (compared to day 0) were determined by a similar modified DFR-like permutation method with Westfall-Young max-T correction. Representative GUCY2C non-responders (**a** and **b**) and responders (**c** and **d**) are shown. All other patient responses are shown in Additional file 1: Figure S1

In preclinical studies, immunization with Ad5-GUCY2C-PADRE induced time- and dose-dependent GUCY2C-specific T-cell and B-cell responses and antitumor immunity mediated by CD8^+^ T cells [17–20, 26]. Here, GUCY2C-specific immune responses were quantified after Ad5-GUCY2C-PADRE administration by ELISA and IFNγ-ELISpot to quantify antibody and T-cell responses, respectively (Additional file 1: Table S2). T-cell responses to PADRE and Ad5 also were quantified by IFNγ-ELISpot. Patient responses typically followed 1 of 4 patterns and representative responses of each are shown in Fig. 2. All other patient responses are shown in Additional file 1: Figure S1. Patient 1001 had no pre-vaccine antibody responses to GUCY2C or T-cell immunity to GUCY2C, PADRE, or Ad5 and Ad5-GUCY2C-PADRE vaccination did not induce responses to these antigens (Fig. 2a). Similarly, while no pre-vaccination responses were observed in patient 1009, vaccination induced Ad5-specific T-cell responses, but not GUCY2C-specific or PADRE-specific responses (Fig. 2b). In contrast to these patients, patient 1008 possessed Ad5-specific T-cell responses prior to vaccination, and Ad5-GUCY2C-PADRE vaccination increased those responses (Fig. 2c). Similarly, GUCY2C-specific T-cell responses also were induced by Ad5-GUCY2C-PADRE vaccination, initially peaking on day 30, followed by a gradual decline through the final 180-day time-point (Fig. 2c). However, this patient produced no PADRE-specific T-cell response or GUCY2C-specific antibody response, recapitulating preclinical studies in mice in which GUCY2C-specific antibody responses require responses to exogenous CD4^+^ “helper” T-cell epitopes, reflecting GUCY2C-specific CD4^+^ T-cell tolerance [17–20, 26]. Patient 1007 was the only patient that produced a response by all three arms of adaptive immunity (Fig. 2d). That patient produced a PADRE-specific CD4^+^ T-cell response, a GUCY2C-specific antibody response, and a GUCY2C-specific CD8^+^ T-cell response that peaked between days 30 and 90, before declining over the remainder of the study.

### GUCY2C-specific T-cell responses are exclusively CD8^+^ T-cell

**Fig. 3:**
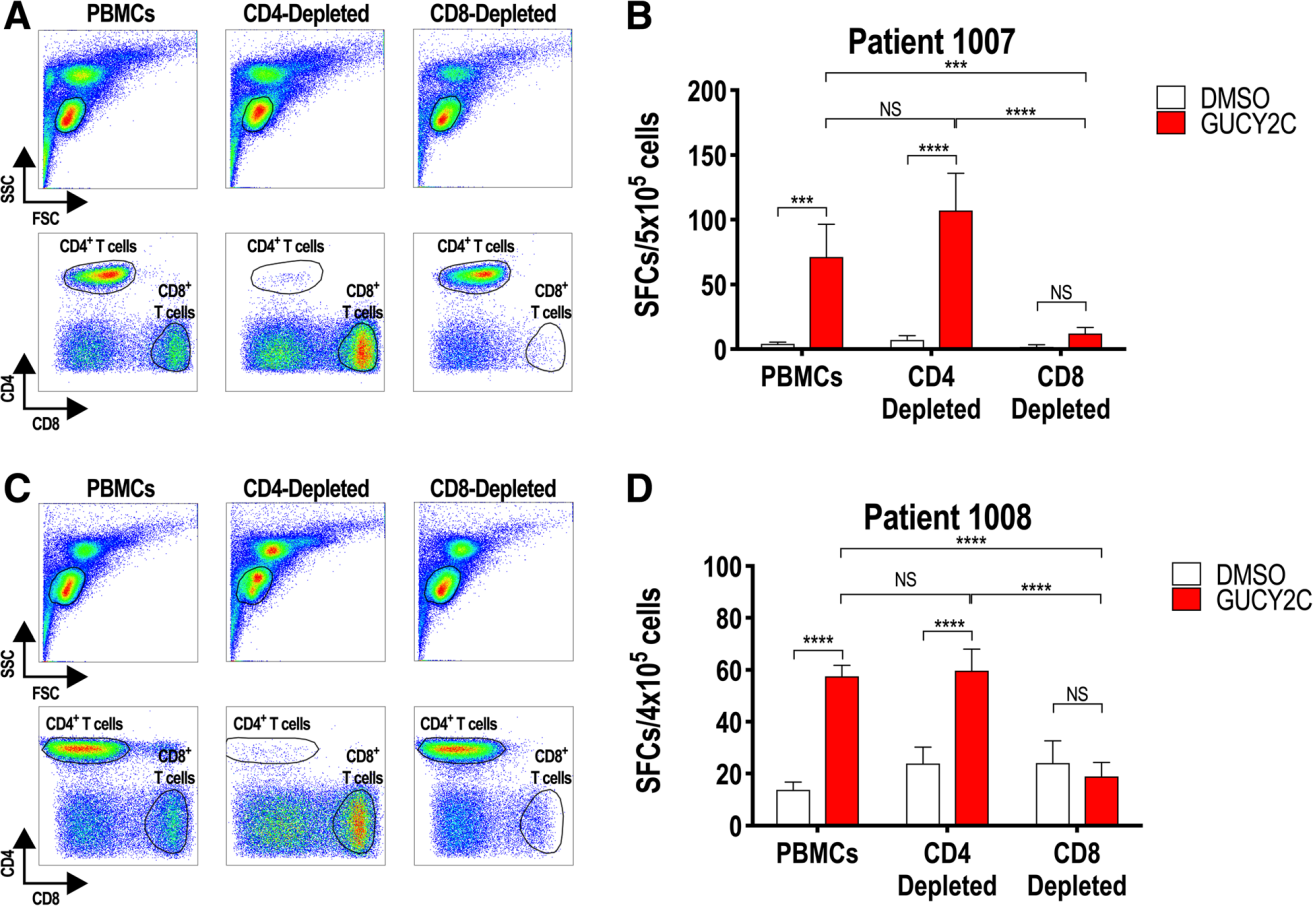
GUCY2C-specific CD8^+^, but not CD4^+^, T-cell responses. PBMCs from GUCY2C-responder patients 1007 (**a** and **b**) and 1008 (**c** and **d**) collected 30 days after Ad5-GUCY2C-PADRE administration were left unsorted or depleted of CD4^+^ or CD8^+^ T cells by MACS. PBMCs, CD4-depleted PBMCs, and CD8-depleted PBMCs were analyzed by FACS to confirm depletion (**a** and **c**) and tested for GUCY2C-specific T-cell responses by IFNγ-ELISpot (**b** and **d**). NS = not significant, *** *P* < 0.001, **** *P* < 0.0001, Two-way ANOVA. Depletion efficiencies determined by FACS were > 98% for CD4^+^ T cells and ~ 75–95% for CD8^+^ T cells

Preclinical studies in mice revealed split tolerance to GUCY2C, eliminating CD4^+^ T cells, but not CD8^+^ T or B cells, which could be fully engaged with exogenous CD4^+^ helper T-cell epitopes (S1 or PADRE) to produce antitumor immunity without autoimmunity [18–20]. To extend that observation from mice to humans, CD4^+^ or CD8^+^ T cells were depleted from PBMCs of patients 1007 and 1008 (GUCY2C responders, Fig. 2), prior to quantification of T-cell responses by IFNγ-ELISpot to determine the cell type responsible for GUCY2C-specific responses (Fig. 3). Depletion of CD8^+^, but not CD4^+^, T cells (Fig. 3a, c) eliminated ELISpot responses in both patients (Fig. 3b, d). Thus, vaccine responses in colon cancer patients are mediated exclusively by CD8^+^ T cells, recapitulating GUCY2C immunology in mice [18–20]. Indeed, selective CD4^+^ T-cell tolerance appears to be a universal mechanism regulating GUCY2C-specific immunity, eliminating GUCY2C-specific CD4^+^ T cells, but not B cells or CD8^+^ T cells, in C57BL/6 [17, 19, 20] and BALB/c [18, 20, 26] mice and in humans (Figs. 2 and 3).

### Ad5 neutralizing antibodies may limit Ad5-GUCY2C-PADRE immunogenicity in patients

**Fig. 4:**
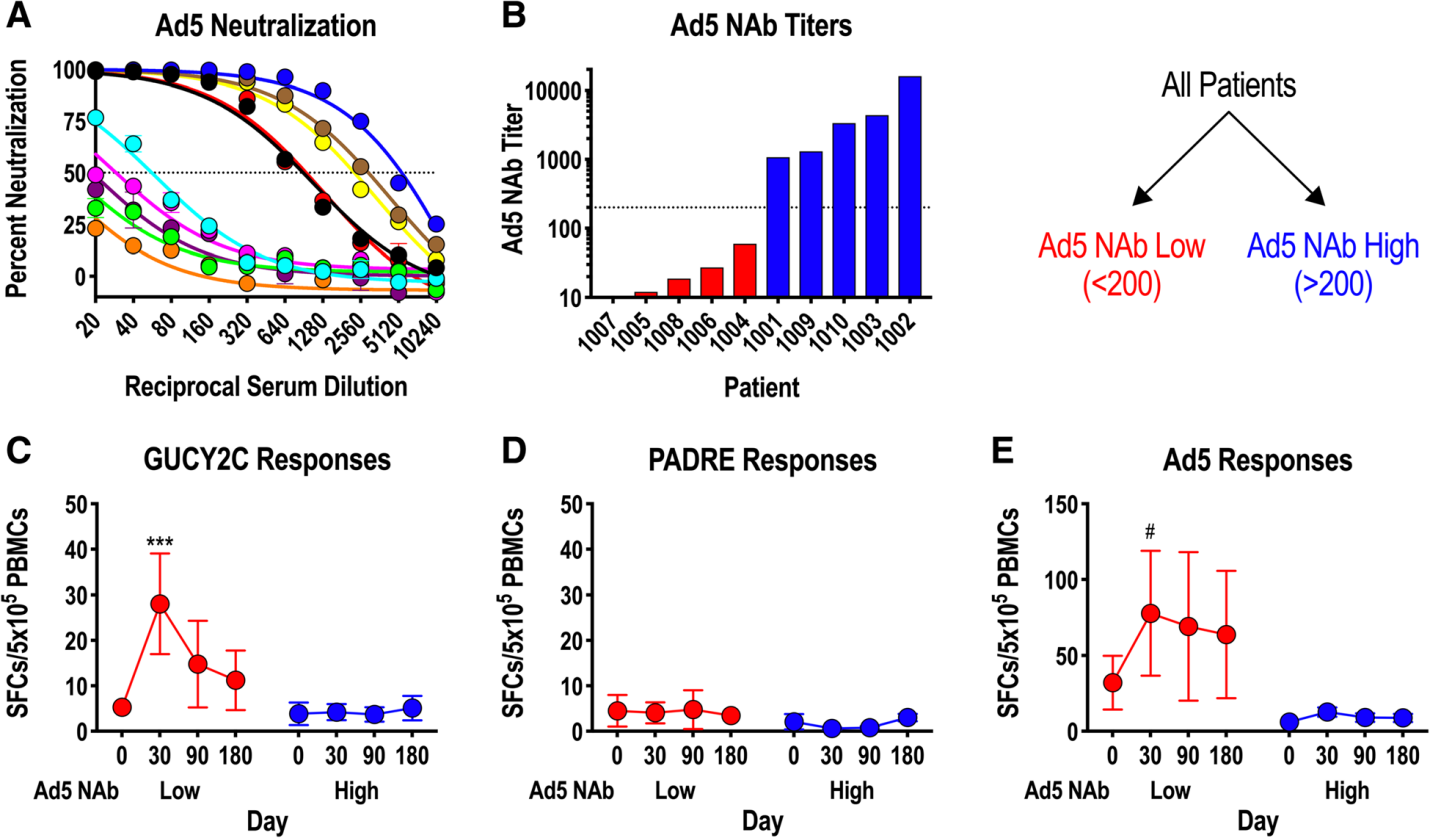
Ad5 neutralizing Abs limit GUCY2C responses in humans**. a**-**e**) patient serum samples collected prior to vaccination (day 0) were analyzed for Ad5 neutralizing antibodies (NAbs) employing an in vitro Ad5-GFP reporter virus inhibition assay (**a**). The Ad5 NAb titer was calculated as the dilution of serum that produced 50% inhibition of GFP reporter expression. **b** patients were rank-ordered by Ad5 NAb titers and patients with titers < 200 were designated as Ad5 NAb Low, while those with titers > 200 where designated Ab5 NAb High (dotted line indicates a titer of 200). **c**-**e** antigen-specific responses to GUCY2C (**c**), PADRE (**d**), and Ad5 (**e**) were compared in Ad5 NAb Low and High patient populations by IFNγ-ELISpot (mixed effect model). # *P* = 0.052, *** *P* < 0.001

Adenovirus, including serotype 5 (Ad5) used in Ad5-GUCY2C-PADRE, is a natural pathogen producing mild infections in humans. Natural exposures induce Ad5-neutralizing antibodies (NAbs) that inhibit future infections or gene delivery by recombinant adenoviruses, including Ad5-based vaccines, by preventing infection of host cells required for antigen expression and induction of immune responses [31–34]. To determine the impact of Ad5 NAbs on Ad5-GUCY2C-PADRE immunogenicity, Ad5 NAbs were quantified in patient serum collected prior to Ad5-GUCY2C-PADRE vaccination (day 0) using an Ad5-GFP reporter virus inhibition bioassay (Fig. 4a). Titers ranged from < 10 to > 10,000 and an obvious pattern emerged in which 50% of the patients had titers below 200 (Ad5 NAb Low) and the other 50% were characterized by titers above 200 (Ad5 NAb High; Fig. 4b). Separating patients into Ad5 NAb Low and High cohorts revealed a relationship between Ad5 NAb titer and GUCY2C-specific T-cell responses in which responses were significantly greater in Ad5 NAb Low patients (Fig. 4c). PADRE-specific T-cell responses, which were generally low, showed no relationship to Ad5 NAb titer (Fig. 4d). Similar to GUCY2C-specific T-cell responses (Fig. 4c), Ad5-specific T-cell responses also were limited in the Ad5 NAb High group (Fig. 4e).

### Mouse models confirm Ad5 NAb sensitivity of Ad5-GUCY2C vaccines

**Fig. 5:**
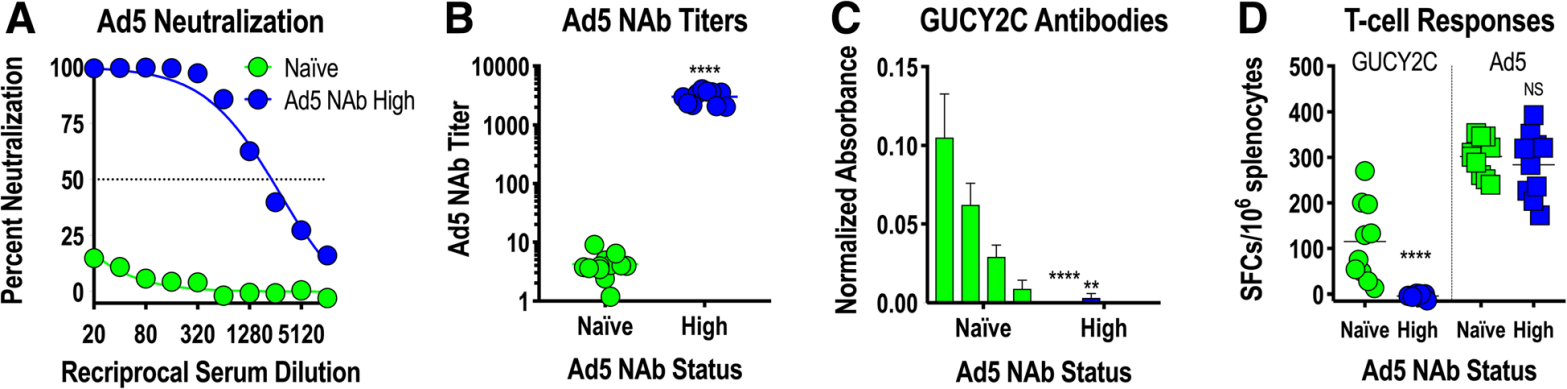
Ad5 neutralizing antibodies limit GUCY2C responses in mice. BALB/c mice were naïve or preconditioned by immunizing 2 times with 10^8^ IFU control Ad5 to induce High Ad5 neutralizing antibody (NAb) titers (*n* = 10 mice/group). **a**, **b** two weeks after preconditioning, serum was collected and an in vitro assay quantifying inhibition of A549 cell infection by an Ad5-GFP reporter virus in the presence of serum titrations (**a**) was used to calculate Ad5 NAb titers (**b**). The Ad5 NAb titer was calculated as the dilution of serum that produced 50% inhibition of GFP reporter expression. **c**, **d** mice were then immunized with 10^8^ IFU of Ad5-mGUCY2C-S1 expressing mouse GUCY2C fused to the CD4^+^ T-helper cell epitope S1. GUCY2C-specific antibody (**c**) and CD8^+^ T-cell responses (**d**) were quantified two weeks later by ELISA and IFNγ-ELISpot, respectively. Bars in (**c**) indicate serum dilutions of 1/20, 1/40, 1/80, and 1/160 (left to right). NS = not significant, ** *P* < 0.01, **** *P* < 0.0001, T-test (**b**) or Two-way ANOVA (**c** and **d**)

To confirm the impact of Ad5 NAbs on GUCY2C vaccination with Ad5 vectors in a mouse model, mice were exposed to control Ad5 vector by two intramuscular immunizations, producing animals with high Ad5 NAb titers (~ 3000; Fig. 5a and b). Ad5-naïve mice or mice with high Ad5 NAb titers were then immunized with Ad5-GUCY2C-S1 (a mouse GUCY2C vaccine analogous to Ad5-GUCY2C-PADRE [18]), and GUCY2C-specific antibody (Fig. 5c) and CD8^+^ T-cell responses (Fig. 5d) were quantified. Consistent with previous mouse studies [31] and human responses to Ad5-GUCY2C-PADRE (Fig. 4), pre-existing Ad5 NAbs eliminated GUCY2C-specific antibody (Fig. 5c) and CD8^+^ T-cell responses (Fig. 5d) in mice. Together, these data suggest that pre-existing Ad5 NAb immunity eliminates Ad5-GUCY2C-PADRE viral particles in vivo prior to entry into host cells, preventing subsequent gene expression and induction of host immune responses, establishing pre-existing Ad5 immunity as a potential barrier to Ad5-GUCY2C-PADRE vaccination in human populations.

## Discussion

There is a significant unmet need for improved treatment for CRC, the 2nd leading cause of cancer mortality worldwide [35]. Most patients undergo surgical resection with curative intent, but 30–50% of these patients experience recurrence and die, underscoring the clinical opportunity for novel therapeutics to improve survival, especially in patients with stage I-III disease. Given that immunotherapies may be most effective in preventing recurrent metastases in patients with minimal residual disease [6], we identified GUCY2C as a promising vaccine target for secondary CRC prevention [16–20]. Here, we translated that paradigm to a first-in-man study examining the safety and immunological efficacy of Ad5-GUCY2C-PADRE in patients with stage I-II colon cancer. Ad5-GUCY2C-PADRE induced antibody and/or T-cell responses directed to GUCY2C in immunized patients, without significant (≥ grade 3) toxicities.

Importantly, GUCY2C-specific T-cell responses involved cytotoxic CD8^+^, but not CD4^+^ helper, T cells, recapitulating results in mice identifying selective CD4^+^ T-cell tolerance as the primary mechanism restricting GUCY2C-specific antitumor immunity [18]. Thus, studies here establish not only the safety and efficacy of Ad5-GUCY2C-PADRE in colorectal cancer patients, but also the importance of “split” tolerance (elimination of CD4^+^ helper, but not CD8^+^ cytolytic T or B cells) as a mechanism shaping immune responses to self antigens in humans. These observations directly impact GUCY2C vaccine design, potentially implicating PADRE as a poor provider of CD4^+^ T-cell help in viral vaccines. PADRE is a synthetic CD4^+^ T-cell epitope which binds most HLA class II molecules and safely induces CD4^+^ T-cell responses in patients when administered in DNA [36], dendritic cell [37], and peptide [38] immunizations. However, results here suggest that PADRE may be poorly immunogenic in the context of adenoviral vaccines, necessitating improved delivery of exogenous CD4^+^ helper T-cell responses for GUCY2C vaccination. While GUCY2C-specific Th1 effector CD4^+^ T-cell responses are absent in mice [18–20] and humans (Fig. 3), mechanisms underlying their loss have not been defined. These may include deletion, anergy, FoxP3+ regulatory (Treg) induction, or others, creating additional opportunities to enhance Ad5-GUCY2C-PADRE efficacy (Treg depletion, for example). In that context, a TCR “retrogenic” mouse model was recently developed to explore mechanisms underlying GUCY2C-specific CD4^+^ T-cell tolerance and solutions to overcome tolerance [39], potentially providing alternatives to incorporation of PADRE to elicit GUCY2C-specific immunity.

Beyond GUCY2C, other self antigens are characterized by split tolerance eliminating CD4^+^ T-cell help, while preserving functional cytolytic CD8^+^ T cells. In mice, the melanosomal antigen Trp2 (tyrosinase-related protein 2) and the growth factor receptor Her2 elicit CD8^+^, but not CD4^+^, T-cell responses. However, provision of exogenous CD4^+^ T-cell help elicited robust cytolytic CD8^+^ T-cell responses, CD8^+^ memory T-cell responses, and antitumor immunity [18]. In that context, enhancing CD4^+^ T-cell help in vaccines targeting these and other self antigens, could substantially improve therapeutic efficacy.

GUCY2C protein (> 200 specimens) and/or mRNA (> 900 specimens) is present in nearly all primary and metastatic human colorectal tumors, regardless of anatomical location or grade [7, 11–13, 40–44], and is over-expressed by > 80% of colorectal tumors [40, 45, 46]. Beyond CRC, GUCY2C is ectopically expressed in approximately 60% of pancreatic, gastric, and esophageal cancers [47–50]. Thus, Ad5-GUCY2C-PADRE may benefit not only CRC patients, but also patients with gastroesophageal and pancreatic cancers, which are typically fatal. Indeed, ~ 25% of all cancer-related deaths in the U.S result from malignancies that may express GUCY2C and which may be treated with GUCY2C-targeted therapies [35].

In conclusion, Ad5-GUCY2C-PADRE is a promising immunotherapeutic for CRC patients with minimal residual disease (stage I-III), as well as patients with gastric, esophageal, and pancreatic cancers. Ad5-GUCY2C-PADRE elicited antibody and cytotoxic T-cell responses in patients following a single administration, without toxicity. Importantly, as in mice [18], split tolerance selectively involving CD4^+^ T cells is a primary mechanism limiting cancer vaccine efficacy in humans that may be exploited to safely elicit antitumor immunity. Thus, optimal provision of CD4^+^ T-cell help may be critical to fully engage self antigen-specific CD8^+^ T and B cells and produce meaningful antitumor immunity and clinical efficacy with cancer vaccines targeting GUCY2C and other self antigens.

## Additional file

Additional file 1: **Table S1.** Baseline characteristics of CRC patients treated with Ad5-GUCY2C-PADRE. **Table S2.** Summary of immune responses to Ad5-GUCY2C-PADRE. **Figure S1.** Ad5-GUCY2C-PADRE-induced immune responses. (PDF 706 kb)
