# Supplementary material for: Split tolerance permits safe Ad5-GUCY2C-PADRE vaccine-induced T-cell responses in colon cancer patients
Source: J Immunother Cancer. 2019 Apr 23;7:104. doi: 10.1186/s40425-019-0576-2 (PMC6477737; doi:10.1186/s40425-019-0576-2)
Supplement: Supplementary file 1 — Table S1. Baseline characteristics of CRC patients treated with Ad5-GUCY2C-PADRE. Table S2. Summary of immune responses to Ad5-GUCY2C-PADRE. Figure S1. Ad5-GUCY2C-PADRE-induced immune responses. (PDF 706 kb) [file 40425_2019_576_MOESM1_ESM.pdf]

Additional File 1 for

Split Tolerance Permits Safe Ad5-GUCY2C-PADRE Vaccine-Induced T-cell Responses in Colon Cancer Patients

Adam E. Snook[1*], Trevor R. Baybutt[1], Bo Xiang[1], Tara S. Abraham[1], John C. Flickinger Jr.[1], Terry Hyslop[2], Tingting Zhan[1], Walter K. Kraft[1], Takami Sato[3], and Scott A. Waldman[1*]

Departments of [1]Pharmacology and Experimental Therapeutics and [3]Medical Oncology, Thomas Jefferson University, Philadelphia, PA 19107

[2]Department of Biostatistics and Bioinformatics, Duke Cancer Institute, Duke University, Durham, NC 27710

This file includes:

Supplementary Table 1. Baseline characteristics of CRC patients treated with Ad5-GUCY2C-PADRE	**Page 1**
Supplementary Table 2. Summary of immune responses to Ad5-GUCY2C-PADRE	**Page 2**
Supplementary Figure 1. Ad5-GUCY2C-PADRE-induced immune responses	**Page 3**

Supplementary Table 1. Baseline characteristics of CRC patients treated with Ad5-GUCY2C-PADRE

**Baseline Characteristic**	**(n = 10)**
Median age (range)	65 (49-76)
Male (%)	5 (50%)
Race	
Caucasian (%)	8 (80%)
African American (%)	2(20%)
TNM Staging	
Stage I (%)	9 (90%)
Stage II (%)	1 (10%)

Supplementary Table 2. Summary of immune responses to Ad5-GUCY2C-PADRE

Patient	Peak GUCY2C Antibody Titer[a]	Antigen-Specific T-Cell Response[b] at Day 30, Significance[c]				Ad5 NAb Titer[a,d]	Ad5 NAb Status[d]
		GUCY2C		PADRE			
1001	20	0.00	*P* = NS	0.00	*P* = NS	1074	High
1002	ND[e]	8.67	*P* = NS	0.42	*P* = NS	15951	High
1003	ND	2.00	*P* = NS	0.50	*P* = NS	4379	High
1004	ND	18.00	*P* < 0.001	11.50	*P* < 0.001	60	Low
1005	ND	10.50	*P* = NS	0.00	*P* = NS	12	Low
1006	ND	6.00	*P* = NS	1.25	*P* = NS	27	Low
1007	640	43.25	*P* < 0.001	7.50	*P* < 0.001	10	Low
1008	ND	63.83	*P* < 0.001	0.00	*P* = NS	19	Low
1009	ND	2.17	*P* = NS	0.33	*P* = NS	1309	High
1010	20	8.17	*P* < 0.001	1.75	*P* = NS	3355	High

[a] *Reciprocal dilutions are shown for GUCY2C antibody and Ad5 NAb titers*
[b] *SFCs/5x10^{5} PBMCs*
[c] *mDFR(2x) compared to background and day 0.*
d Ad5 NAb titers and status prior to Ad5-GUCY2C-PADRE administration are shown
[e] *ND = not detected*

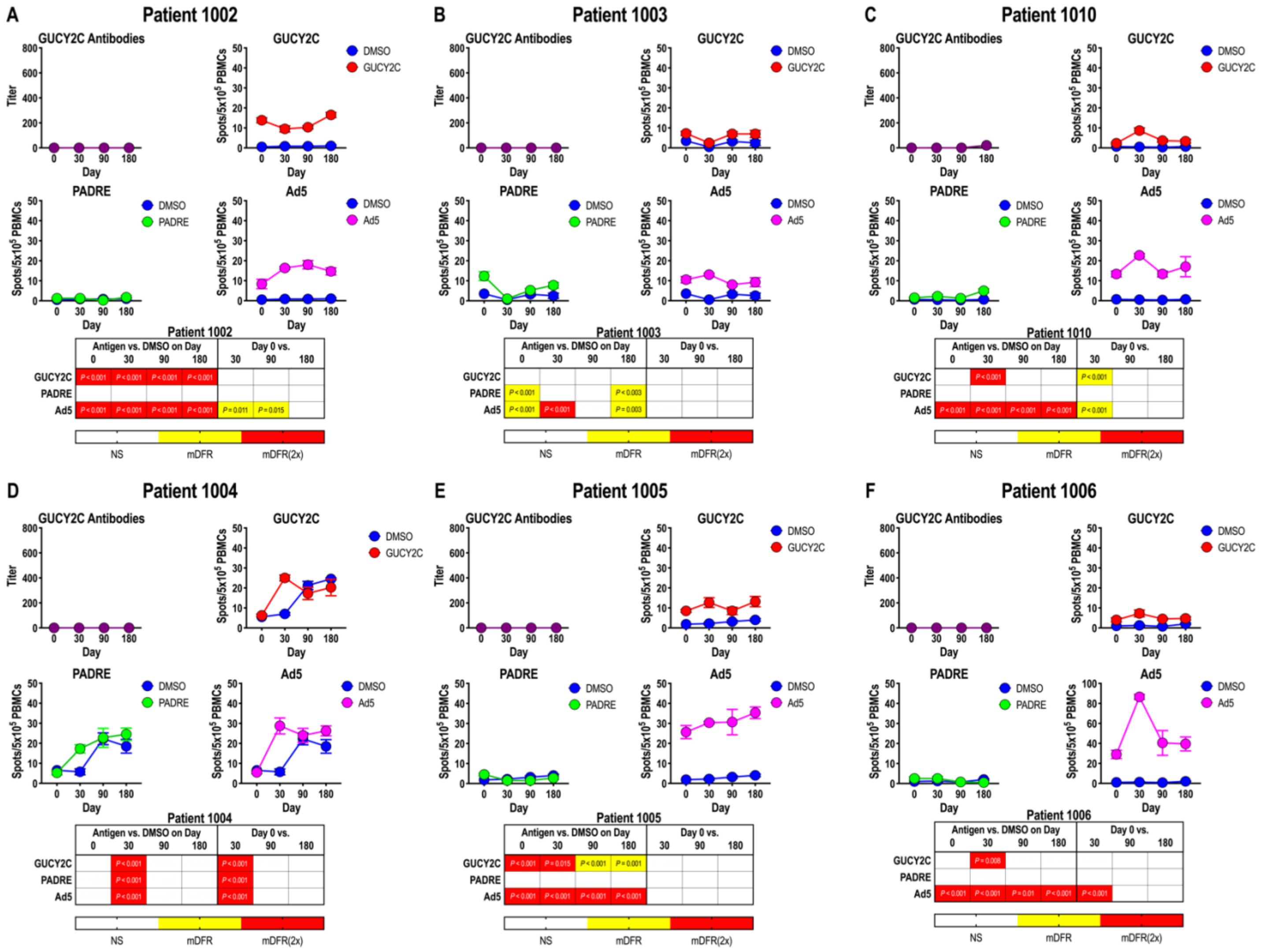

Figure 1. Ad5-GUCY2C-PADRE-induced immune responses. Patient blood samples were collected before (day 0) and 30, 90 and 180 days after Ad5-GUCY2C-PADRE immunization. GUCY2C-specific antibody titers were quantified by ELISA and GUCY2C, PADRE, and Ad5 -specific T-cell responses were quantified by IFNγ-ELISpot. ELISpot assays employed DMSO as an antigen-negative control. The statistical significance for T-cell responses at each time point (compared to DMSO) was determined by modified DFR(eq) or DFR(2x) after Westfall–Young max-T correction, and p-values <5% are shown in yellow [mDFR(eq)] or red [mDFR(2x)], respectively. The statistical significance of T-cell responses obtained for each post-vaccination time point (compared to day 0) were determined by a similar modified DFR-like permutation method with Westfall-Young max-T correction. Representative GUCY2C non-responders and responders are shown in **Figure 2**. All other patient responses are shown here.
